# PET-guided therapy: synergies, strategies, and future directions in external beam radiation therapy and radiopharmaceutical therapy

**DOI:** 10.3389/fmed.2026.1794722

**Published:** 2026-04-15

**Authors:** May Abdel-Wahab, Francesco Giammarile

**Affiliations:** Division of Human Health, Department of Nuclear Sciences and Applications, International Atomic Energy Agency, Vienna, Austria

**Keywords:** external beam radiation therapy, FDG, PET/CT, PET-guided therapy, radiopharmaceutical therapy

## Abstract

Positron Emission Tomography (PET) has evolved from a purely diagnostic modality into a cornerstone of precision radiation oncology. PET now informs patient selection, target delineation, treatment personalization, and post-therapy evaluation across External Beam Radiation Therapy (EBRT) and Radiopharmaceutical Therapy (RPT). Radiotracers provide quantitative data on tumor biology, heterogeneity, receptor expression, and therapeutic response, enabling a shift from morphology-based to biology-driven oncology. PET-guided therapy is increasingly used to select patients for molecular radiotherapy, guide EBRT boost volumes, monitor receptor occupancy, and personalize activity prescription. Combined modality approaches—such as EBRT plus PSMA-RLT in prostate cancer or EBRT plus SSTR-RLT in neuroendocrine tumors—are supported by biological rationales involving synergy between external and internal radiation sources. Adaptive strategies based on mid-treatment PET show promise in improving local control while minimizing toxicity. This review summarizes the current landscape and emerging applications of PET-guided therapy, highlighting methodological synergies between EBRT and RPT, strategies for treatment sequencing, biological dose painting, and adaptive therapy. It provides practical recommendations for implementing PET-guided workflows and discusses advances in radiobiology-informed dosimetry, whole-body PET technologies, and novel imaging biomarkers, including fibroblast activation protein inhibitors (FAPI), as key drivers of innovation. As PET technology evolves toward ultra-low-dose, ultra-fast total-body systems, the role of molecular imaging in therapeutic decision-making is expected to expand, ushering in a new era of biologically guided radiation oncology.

## 1 Introduction

Molecular imaging with Positron Emission Tomography (PET) has undergone a profound transformation over the last two decades: from a purely diagnostic modality to a cornerstone of precision radiation oncology. PET now informs not only staging and response monitoring, but is increasingly used to guide therapeutic decisions—in both External-Beam Radiation Therapy (EBRT) and Radiopharmaceutical Therapy (RPT). This convergence is rooted in several interrelated developments that are reshaping the landscape of cancer treatment (1).

First, PET enables biology-driven treatment planning: by revealing molecular features such as metabolism, receptor expression, hypoxia or stromal activation. Importantly, tracer uptake should not be interpreted as a direct surrogate for a single biological process. For example, FDG accumulation may result not only from increased glycolytic metabolism but also from enhanced vascular delivery, GLUT1 overexpression, or alternative metabolic phenotypes such as anti-Warburg patterns. By identifying dominant tumor subvolumes through tracer-specific transport and retention mechanisms, PET can help delineate “biological target volumes” (BTV), providing a more nuanced basis for radiotherapy than conventional anatomical imaging and potentially improving tumor control while sparing healthy tissue (2, 3).

Second, advances in personalized dosimetry have strengthened the value of PET in RPT. Quantitative PET metrics and tracer kinetics can be used to estimate radiopharmaceutical uptake and predict the absorbed dose in tumors and organs at risk. Dynamic PET/CT combined with Monte Carlo dosimetry models enables voxel-based dose calculation prior to therapy, offering a path toward individualized activity prescription. Indeed, a recent proof-of-concept demonstrated substantial inter-patient variability in absorbed doses when using standard fixed RPT doses, underscoring the need for personalized planning (3).

Third, the complementary mechanisms of EBRT and RPT—external high-dose fractionated radiation vs. internal systemic low-dose-rate irradiation—can be strategically combined or sequenced. PET-guided identification of disease burden, receptor expression, and spatial distribution opens the possibility for combination and sequencing strategies that exploit their synergy for improved therapeutic ratio. For example, RPT may treat micro-metastatic disease while EBRT consolidates macroscopic lesions or vice versa, depending on tumor biology and disease distribution. A recent review of PSMA-PET guided radiotherapy in prostate cancer highlights such synergy and calls for clinical trials to validate these integrated approaches (4).

Fourth, continuous technological advances in imaging are expanding the scope and precision of PET-guided therapy. Wide-axial field-of-view (total-body) PET scanners increase sensitivity, which can allow reductions in injected activity or acquisition time compared with conventional PET systems. However, PET imaging remains substantially slower than CT, and these scanners should not be characterized as “fast” in absolute terms. Consequently, while they improve lesion detectability, enable low-dose whole-body imaging, dynamic acquisitions, delayed imaging windows, and whole-body dosimetry, current PET technology does not yet support routine use for rapid treatment delivery or real-time therapy guidance. Additionally, the development of novel and highly specific radiotracers—such as those targeting fibroblast activation protein (FAP), hypoxia, or tumor-specific receptors beyond PSMA or somatostatin receptors—broadens the therapeutic and diagnostic potential across diverse cancer types (5).

Wide-axial field-of-view (total-body) PET scanners offer increased sensitivity, which can allow reductions in injected activity or acquisition time compared with conventional PET systems. However, PET imaging remains substantially slower than CT, and these scanners should not be characterized as “fast” in absolute terms. Consequently, while they enable low-dose whole-body imaging, dynamic acquisitions, and delayed imaging windows, current PET technology does not yet support routine use for rapid treatment delivery or real-time therapy guidance.

Finally, this evolution is accompanied by a shift toward **outcome-driven adaptive therapy**. Early PET response indicators—including changes in SUV, tracer kinetics or volumetric metrics—can inform adjustments in dose, fractionation or therapeutic modality mid-course, tailoring treatment to each patient's biology. This adaptive paradigm promises to improve efficacy, reduce toxicity, and optimize resource use (6).

In this review, we examine the clinical and translational advances that underpin PET-guided EBRT and RPT, analyze strategies for integrating these modalities, and discuss the radiobiological and dosimetric challenges involved. We highlight leading technical developments—especially total-body PET and novel imaging biomarkers such as FAP-targeting ligands—and explore how they may reshape future treatment paradigms. Finally, we outline opportunities and challenges in implementing PET-guided, outcome-driven radiation therapy in clinical practice, emphasizing the need for standardized protocols, prospective trials, and multidisciplinary collaboration.

## 2 PET-guided therapy

### 2.1 Concept and clinical rationale

PET has become an essential pillar of precision oncology because it provides quantitative and biologically meaningful information that cannot be obtained from anatomical imaging alone. Beyond simple visualization of lesions, PET offers a wide range of functional biomarkers. Standardized uptake values (SUV), kinetic parameters derived from dynamic imaging, and voxel-based quantification correlate with tumor aggressiveness, proliferative activity, metabolic rate, and treatment sensitivity. These quantitative metrics have been shown to predict outcomes across multiple malignancies, supporting their integration into clinical decision-making (7).

Another key advantage of PET is its ability to visualize spatial heterogeneity within tumors. Intratumoral variations in metabolism, hypoxia, proliferative activity, receptor expression, or stromal activation can be identified *in vivo*, revealing biologically distinct subregions that may influence treatment response. PET tracers such as ^18^F-FDG, ^18^F-FMISO, ^18^F-FLT, ^68^Ga-FAPI, or receptor-targeted ligands can highlight these subvolumes with high sensitivity (Table 1). However, interpretation must be cautious: treatment-related changes such as inflammation or radiation-induced necrosis may exhibit transient tracer uptake, and observed PET signal changes may reflect multiple biological processes rather than viable tumor alone (6). Understanding these limitations is essential to guide careful, biology-driven treatment planning while avoiding overestimation of PET-defined tumor activity.

**Table 1:** Key PET tracers for therapy guidance.

Tracer	Biological target	EBRT use	RPT use	Clinical applications
18F-FDG	Glucose metabolism	Target delineation, response assessment, adaptive planning	Diagnostic baseline Therapy follow-up	Wide range of tumors (lung, head & neck, esophageal, lymphoma)
68Ga/18F-PSMA	Prostate-specific membrane antigen	Target delineation, nodal staging	Patient selection for ^117^Lu/225Ac-PSMA therapy, dosimetry	Prostate cancer, recurrent/metastatic disease
68Ga/18F-FAPI	Tumor stroma (fibroblast activation protein)	*Target delineation, boost planning in stroma-rich tumors*	Diagnostic baseline Therapy follow-up *Theranostic*	Pancreatic, breast, cholangiocarcinoma, sarcomas
18F-FLT	Cellular proliferation (thymidine kinase activity)	*Response assessment, proliferation-guided adaptation*	*Therapy follow-up*	Head & neck, glioma, lymphoma
18F-FMISO/18F-FAZA	Hypoxia	*Hypoxia-targeted dose escalation*	*Therapy follow-up*	Head & neck, lung, cervical cancer
89Zr/64Cu- CD8/PD-L1	Immune cell infiltration/checkpoint expression	*Adaptive immuno-radiotherapy planning*	*Radioimmunotherapy*	Immunotherapy combination strategies, tumor microenvironment monitoring

In italic: experimental.

Molecular imaging also enables precise “theranostic match-making,” ensuring that the therapeutic target is present before treatment is initiated. This principle is now central to radionuclide therapy, where PET identifies PSMA expression in prostate cancer, somatostatin receptor expression in neuroendocrine tumors, or FAP expression in stromal-driven malignancies (8, 9). Importantly, while PET confirms target presence and helps determine treatment eligibility, it does not reliably predict therapeutic response. For instance, some patients with PSMA-avid disease may not respond to PSMA-targeted radioligand therapy, highlighting the need for ongoing research into radiohybrid ligands and combination strategies to improve outcomes (10).

The clinical value of PET naturally extends to both EBRT and RPT workflows. In EBRT, PET significantly enhances target delineation by refining gross tumor volume boundaries and identifying regions at risk for microscopic extension. Importantly, PET-based characterization of radioresistant subvolumes—for example hypoxic regions on FMISO-PET—provides the biological foundation for adaptive intensity modulation or biological dose painting strategies (11).

In RPT, PET plays an equally pivotal role by confirming molecular target expression, quantifying lesion uptake, and predicting absorbed dose through pre-therapeutic dosimetry. PET also identifies off-target accumulations in organs at risk, helping to personalize administered activity and minimize toxicity. Several studies have explored correlations between PET-based dosimetry and clinical outcomes, highlighting the potential of PET to inform individualized RPT planning (12). However, limitations remain, including differences between diagnostic tracers and therapeutic agents, variations in biological and physical half-lives, and uncertainties in translating PET-derived activity into accurate absorbed doses. These factors currently constrain the routine clinical application of PET-based dosimetry for therapy planning.

Taken together, PET provides a biologically informed framework for therapeutic decision-making, enabling a shift from anatomy-driven to biology-driven radiation oncology. Its quantitative, spatial, and molecular insights underpin the ongoing integration of PET with both EBRT and RPT and set the stage for fully personalized, adaptive radiation therapy in the near future.

### 2.2 PET-guided EBRT

PET has become increasingly valuable in modern external beam radiotherapy (EBRT), primarily by improving target delineation and informing treatment planning (13). Among the most widely used tracers, ^18^F-FDG has demonstrated substantial value in refining gross tumor volume (GTV) delineation across several malignancies, including lung, head-and-neck, esophageal cancers, and lymphomas. PET-derived metabolic boundaries frequently differ from CT-based contours, reducing geographic miss and enabling more accurate radiation planning (14). However, the concepts of molecular specificity and adaptive EBRT workflows based on repeated PET imaging during therapy are not yet routinely applied clinically and can be confounded by transient inflammatory or post-irradiation changes. Beyond improving anatomic precision, ^18^F-FDG can reveal intratumoral heterogeneity, identifying metabolically active subregions, but these “biological target volumes” (BTV) should be interpreted with caution, as tracer uptake may reflect multiple biological processes beyond viable tumor tissue, including inflammation or treatment-related changes.

A growing body of work has explored PET tracers that characterize specific biological processes relevant to radiosensitivity. Hypoxia imaging with tracers such as ^18^F-FMISO or ^18^F-FAZA can identify regions of low oxygenation, which are generally more radioresistant (15). However, during fractionated radiotherapy, tumor shrinkage and re-oxygenation may alter these initially hypoxic areas, limiting the direct applicability of hypoxia imaging for selective dose escalation. Similarly, ^18^F-FLT PET highlights proliferative tumor compartments and may help stratify aggressive subvolumes, although its clinical integration remains investigational (16). In prostate cancer, the rapid adoption of PSMA PET has markedly improved the precision of EBRT planning by detecting microscopic nodal disease and guiding focal boosts to dominant intraprostatic lesions (17). In the central nervous system, amino-acid PET tracers are particularly useful for differentiating tumor from treatment-related changes, improving GTV definition for high-grade gliomas, especially glioblastoma (18). By contrast, Fluciclovine, originally developed for prostate cancer, is generally less effective for brain tumors, whereas PSMA-targeted tracers are more suitable for prostate applications.

PET also supports adaptive radiotherapy strategies by providing early biological feedback during treatment. Mid-treatment PET responses correlate with overall outcome in several cancers, allowing clinicians to modify radiotherapy plans based on early metabolic changes. Responders may become candidates for de-escalation to reduce morbidity, while persistent PET-avid disease can be selectively boosted with stereotactic or intensity-modulated techniques. Prospective studies have shown the feasibility and safety of PET-adapted boost regimens in head-and-neck and lung cancers, with potential improvements in local control (19).

Emerging technologies further expand the role of PET in EBRT. Biology-guided radiotherapy (BgRT), which uses real-time PET signal from continuously detected annihilation photons to steer dynamic beam delivery, represents a novel and potentially transformative approach. Early preclinical and first-in-human investigations show promising feasibility and suggest that PET-derived signals may 1 day guide dose deposition in real time. Additionally, the development of hybrid PET-linear accelerator systems and workflows for PET-informed stereotactic ablative radiotherapy (SABR) underscores the increasing integration of molecular imaging into radiotherapy delivery (13).

The clinical impact of PET in EBRT extends beyond target definition. Accurate staging with PET prevents futile radical radiotherapy by identifying occult metastatic disease, thereby avoiding toxicity without oncologic benefit. In many tumors, PET-based delineation supports biological dose-painting strategies that escalate dose selectively to PET-avid subvolumes while sparing organs at risk. The combination of initial PET-based planning with mid-treatment metabolic assessment positions molecular imaging as a central tool in adaptive radiotherapy, enabling dynamic replanning that reflects evolving tumor biology rather than static anatomy.

Altogether, PET-guided EBRT represents a paradigm shift toward biologically informed radiation oncology. By integrating metabolic, hypoxic, proliferative, or receptor-specific information into treatment planning and adaptation, PET allows radiation to be tailored more precisely to tumor biology. As technological advances such as total-body PET, BgRT, and PET-LINAC integration mature, the role of molecular imaging in EBRT is expected to become increasingly central to precision radiotherapy.

### 2.3 PET-guided RPT: current status and PET's role

Radiopharmaceutical therapy (RPT) has progressed from a niche modality to an established therapeutic pillar in oncology, exemplified by the success of [^177^Lu]Lu-PSMA-617 in metastatic castration-resistant prostate cancer and [^117^Lu]Lu-DOTATATE peptide receptor radionuclide therapy (PRRT) in neuroendocrine tumors. Its fundamental strength lies in delivering systemic yet highly targeted radiation, enabling simultaneous treatment of multiple lesions while sparing healthy tissues (20). Ongoing developments—including novel ligands, fibroblast activation protein inhibitors (FAPIs), and alpha emitters—are rapidly broadening clinical indications.

PET imaging underpins this therapeutic expansion by integrating diagnosis, patient selection, and quantitative monitoring within a single theranostic paradigm. Companion PET tracers (e.g., PSMA, SSTR, FAPI, and HER2) enable confirmation of molecular target expression, stratification of candidates for therapy, and estimation of tumor burden (21). Quantitative PET additionally provides essential input for individualized dosimetry, which has become increasingly recognized as critical given the large inter-patient variability in absorbed doses when fixed administered activities are used (9).

In clinical practice, PET supports all phases of RPT: verification of target expression prior to therapy; early prediction of tumor and organ absorbed doses through SUV-based or kinetic modeling approaches; protection of dose-limiting organs such as kidneys, bone marrow, and salivary glands (notably in PSMA-directed RLT); and post-therapy assessment of treatment response. As dosimetry techniques mature—extending from organ-level to voxel-wise methods—the field is moving toward fully quantitative PET-based planning analogous to paradigms long established in external beam radiotherapy. This evolution is expected to enable more accurate, personalized activity prescriptions and improved clinical outcomes (9).

## 3 Synergies between PET, EBRT, and RPT

### 3.1 Biological rationale

External beam radiotherapy (EBRT) and radiopharmaceutical therapy (RPT) deliver ionizing radiation through fundamentally different spatial and temporal profiles, creating opportunities for genuine therapeutic synergy. EBRT provides high-dose, externally administered radiation with millimetric precision and overall dose homogeneity, while RPT delivers low-to-medium radiation doses systemically through radiolabeled ligands whose biodistribution follows tumor biology, resulting in heterogeneous but biologically targeted dose deposition (22).

These complementary characteristics support several synergistic interactions. EBRT can reduce bulky disease or act as a radiosensitizer, enhancing the effectiveness of subsequent RPT. Conversely, RPT can target microscopic or disseminated lesions that lie outside EBRT treatment fields. PET imaging integrates these modalities by defining tumor biology, mapping target expression, and identifying subregions that may benefit from intensified local or systemic treatment. PET thereby facilitates rational sequencing strategies and enables biologically informed, patient-specific planning (23).

### 3.2 Clinical and emerging examples of synergy

The prostate cancer setting offers one of the clearest illustrations of this interaction. PSMA PET is highly effective for identifying intraprostatic lesions and mapping systemic disease in patients with prostate cancer. Its primary role is in accurate staging and target localization for radiotherapy planning, rather than directly guiding EBRT dose escalation or indicating concurrent [^117^Lu]Lu-PSMA therapy, which is generally reserved for patients with metastatic or treatment-refractory disease (24).

In neuroendocrine tumors, SSTR PET maps somatostatin receptor–expressing lesions that may benefit first from EBRT boosts, particularly for symptomatic or threatening sites, before administration of [^117^Lu]Lu-DOTATATE PRRT. This combined approach is supported by the PRRT evidence base, notably the NETTER-1 trial (20).

Head and neck cancers represent another area of interest: FDG PET–guided EBRT dose escalation has improved locoregional control in several studies, and conceptual frameworks now explore the integration of RPT to address hypoxic or radioresistant regions—an approach still in early clinical development (25).

Several emerging fields are expanding the landscape further. FAPI PET enables visualization of tumor stroma and may guide EBRT boosts or identify candidates for future FAPI-based RPT (26). Exploratory early-phase studies investigating whether high-LET alpha-emitters radiopharmaceuticals could complement EBRT in selected resistant lesions (27). However, in the context of oligometastatic disease, stereotactic body radiotherapy (SBRT) remains the standard local treatment, and these combination strategies are currently investigational and not part of routine clinical practice (28). Moreover, PET-informed sequencing strategies—administering RPT before EBRT to reduce systemic burden or conversely using EBRT upfront to modulate tumor vasculature and microenvironment—are being actively evaluated (29).

While PET-guided adaptive radiotherapy has been investigated in research settings (30), repeated tracer injections during treatment and treatment-related inflammatory changes can complicate the interpretation of metabolic signals. Therefore, EBRT plans are not routinely modified based on metabolic changes in current clinical practice.

Although clinical evidence for combined EBRT–RPT strategies remains preliminary, early data suggest that these integrated approaches may enhance tumor control without compromising safety in carefully selected scenarios. Ongoing trials are now testing these concepts in prostate cancer, neuroendocrine tumors, and other solid malignancies, with PET imaging serving as the unifying tool to guide patient selection, sequencing, and adaptive refinement of both modalities.

## 4 Practical strategies for PET-guided treatment planning

PET imaging plays an important role in the design and optimization of combined RPT and EBRT. Its primary clinical utility lies in patient staging and informing treatment decisions, rather than routine use for sequencing strategies or adaptive radiotherapy workflows.

### 4.1 PET-informed patient selection

Patient eligibility for targeted therapies relies heavily on PET-based biomarkers. Quantitative thresholds such as SUVmax have been shown to predict therapeutic benefit—for example, high PSMA uptake (SUVmax ≥ 10) correlates with improved response to [^117^Lu]Lu-PSMA treatment, while low tumor-to-salivary-gland ratios may forecast higher xerostomia risk (31). Beyond simple uptake values, PET also reveals intratumoural heterogeneity, which has been linked to differential treatment sensitivity and may help distinguish responders from non-responders. This capacity to map biological variability is becoming increasingly relevant as trials explore individualized RPT dosing and EBRT dose escalation based on PET-defined subregions (32).

### 4.2 Treatment sequencing strategies

Optimizing the sequence of RPT and EBRT relies on understanding how systemic and local radiation interact within tumor and normal tissues. PET provides the biological guidance required for these decisions. Administering RPT first may shrink systemic burden and improve EBRT geometry, whereas EBRT upfront can secure local control and modulate tumor microenvironmental factors that influence radioligand uptake. More complex strategies—such as intercalating EBRT between cycles of RPT or using simultaneous integrated boosts guided by PET-defined biological subvolumes—are being actively explored (13). PET monitoring throughout treatment allows clinicians to evaluate biological changes over time, adapting the sequence or intensity of each modality as needed (33).

### 4.3 Biological target volume (BTV) delineation and dose painting

PET imaging can complement anatomical delineation by providing biologically informed targeting for radiation planning. Conceptual frameworks such as “dose painting by contours” (defining PET-positive subvolumes within tumors) and “dose painting by numbers” (mapping voxel-wise PET intensity values onto corresponding radiation dose) levels illustrate potential strategies for biologically guided radiotherapy. However, these approaches remain largely investigational: clinical adoption is limited, and PET uptake should not be interpreted as a direct surrogate for absorbed dose in radioligand therapy. Accurate PET–CT co-registration, quantification, and standardization across imaging systems are essential but do not yet fully address these uncertainties. Clinical experience from FDG-guided dose escalation in head and neck cancer illustrates the potential of this strategy to enhance control in biologically aggressive regions (34).

### 4.4 Integrated workflow: from patient selection to toxicity monitoring

The construction of effective PET-guided multimodal therapies necessitates cohesive coordination between nuclear medicine, radiation oncology, medical physics, and dosimetry teams. High-sensitivity tracers such as PSMA, SSTR analogs, FAPI, or FDG allow comprehensive staging and identification of candidates for consolidation with EBRT or systemic RPT. PET-based quantification underpins personalized dosimetry, assisting with activity selection in RPT and enabling harmonization with EBRT dose constraints, particularly for marrow, renal tissue, and salivary glands. Serial or mid-treatment PET—including emerging total-body PET platforms—also supports adaptive workflows by assessing early metabolic responses and refining EBRT target volumes or RPT planning accordingly (35).

Safety remains a key consideration in combined-modality approaches. However, PET-based organ uptake should not be interpreted as a direct surrogate for absorbed dose in RPT, due to differences between diagnostic tracers and therapeutic radiopharmaceuticals (e.g., isotope size, biological distribution, and half-life). In EBRT, dose planning is based on CT-defined anatomical structures, and discrepancies between PET uptake and CT anatomy can further complicate interpretation (36).

Together, these strategies demonstrate how PET functions not merely as a diagnostic tool but as a unifying framework enabling biologically precise, personalized planning of combined EBRT–RPT treatments.

## 5 Radiobiology and dosimetry

Radiopharmaceutical therapy (RPT) and external beam radiotherapy (EBRT) deliver radiation according to fundamentally different physical and biological principles, making accurate dosimetry essential for the safe and effective integration of both modalities. While EBRT delivers high-dose radiation in short fractions, RPT administers radiation continuously at low dose rates over several days, governed by the pharmacokinetics and biodistribution of the radiolabeled compound. This prolonged exposure allows ongoing DNA repair processes to occur during irradiation, altering biological effectiveness and limiting the applicability of traditional EBRT-based radiobiological metrics without model adjustments.

PET imaging plays a central role in RPT dosimetry because time-integrated activity—derived from PET- or SPECT-based kinetic measurements—determines the absorbed dose delivered to tumors and normal tissues. The dose distribution in RPT is inherently heterogeneous, reflecting biological uptake patterns, and becomes particularly complex with alpha emitters, whose high-linear energy transfer (LET) radiation produces dense ionization clusters and highly localized cytotoxicity at the microscale. As a result, microdosimetric analyses and advanced radiobiological formalisms, including generalized linear-quadratic (gLQ) models, have been proposed to better predict tumor control and normal tissue effects in RPT and combined-modality treatments (37).

Voxel-wise dosimetry methods, increasingly enabled by quantitative PET, support highly individualized treatment planning by mapping spatial heterogeneity in absorbed dose and linking it with biological response. This is especially relevant for organs-at-risk such as kidneys, bone marrow and salivary glands, which often limit activity escalation. Across multiple RPTs—including [^117^Lu]Lu-DOTATATE and [^117^Lu]Lu-PSMA—studies have shown wide, reinforcing the evidence that fixed, “one-size-fits-all” activity schemes are suboptimal (38).

These considerations are even more critical when EBRT and RPT are combined. The superposition of high-dose-rate, spatially conformal EBRT with the biologically driven, prolonged low-dose-rate exposure of RPT creates complex dose–effect relationships that cannot be adequately captured using EBRT-based radiobiological assumptions alone. Accurate, patient-specific dosimetry becomes indispensable not only for predicting cumulative organ toxicity but also for designing rational activity levels, sequencing strategies, and dose constraints that exploit synergy without exceeding biological limits (39).

By integrating quantitative imaging, radiobiological modeling, and voxel-based dose assessment, PET-guided dosimetry is evolving into a cornerstone of personalized therapy. It provides the foundation for optimizing combined EBRT–RPT strategies, improving tumor control while preserving safety, and paving the way toward biologically informed, adaptive, and truly individualized radiotherapy (3).

## 6 Recent advances

### 6.1 Technical developments

The advent of total-body PET (TB-PET) has marked a significant leap forward in molecular imaging. By extending the axial field-of-view so as to cover the entire body in a single bed position, TB-PET scanners dramatically increase system sensitivity and signal-to-noise ratio: up to ~40-fold compared to conventional PET/CT systems (40). This gain in sensitivity enables ultra-low-dose imaging while preserving high image quality, making repeated longitudinal scans feasible with minimal radiation exposure (41). Moreover, TB-PET may improve quantitation of tracer uptake over time: a key asset for radiopharmaceutical therapy (RPT) dosimetry and biologically adaptive radiotherapy planning (40). Indeed, in a study of liver radioembolization (^90^Y PET), the use of a total-body scanner resulted in more accurate dose quantification, with narrower dose distribution tails and lower image noise compared to conventional PET/CT (42).

In addition to hardware advances, progress in image processing and analysis is contributing to enhanced PET-based workflows. Improvements in auto-segmentation, novel reconstruction algorithms (including AI-assisted methods), and ongoing standardization initiatives by professional bodies (e.g., for radiomics and parametric imaging) are being developed to translate raw PET data into reliable quantitative biomarkers. Combined with the ultra-sensitive detection capabilities of TB-PET, these advances open the door to quantitative PET biomarkers—including SUV metrics, volumetrics, texture features, and radiomics signatures—that may help predict radiation response, guide adaptive therapy strategies, and identify early non-responders. However, their successful implementation will require prospective validation and standardization across centers (43).

### 6.2 Imaging biomarkers

One of the most promising developments in PET imaging biomarkers is the use of tracers targeting the stromal compartment of tumors rather than metabolic activity *per se*. In particular, fibroblast activation protein inhibitor (FAPI) PET has emerged as a highly sensitive method to visualize cancer-associated fibroblasts (CAFs), which are present in the tumor microenvironment and often constitute a substantial portion of the tumor mass (21).

FAPI tracers typically exhibit rapid uptake in lesions, minimal background signal in normal tissues, and consequently high tumor-to-background ratios—advantages that make them particularly useful for detecting tumors with low glycolytic activity, or tumors located in regions with high physiologic uptake (e.g., liver and brain) where conventional FDG PET performs poorly (44). In several malignancies, including low-grade sarcomas, FAPI PET/CT has demonstrated higher detection rates and accuracy than standard ^18^F-FDG PET. For instance, in a cohort of patients with intermediate or low-grade sarcomas, ^68^Ga-FAPI PET detected lesions with significantly greater sensitivity and identified almost half of patients as candidates for FAP-targeted radiopharmaceutical therapy (FAP-RPT) (45).

Beyond detection, FAPI PET is attractive in the context of combined radiotherapy and radionuclide therapy (RLT), or theranostic strategies: the same FAPI molecule may be radiolabeled with therapeutic radionuclides (e.g., ^117^Lu and ^225^Ac), enabling a seamless transition from imaging to treatment (21). The favorable pharmacokinetics (rapid clearance from non-target tissue, short injection-to-scan interval and low radiation burden) further support its practicality in clinical workflows.

In addition to FAPI, other PET biomarkers are being explored to capture different aspects of tumor biology: immune-PET tracers (e.g., targeting PD-L1 or CD8) may allow visualization of immune contexture relevant for immuno-radiotherapy; proliferation tracers such as FLT; and hypoxia tracers (e.g., FMISO, FAZA) may inform on radioresistant tumor regions. Integrating these diverse biomarkers with advanced PET technologies promises a rich toolbox for truly personalized radiation therapy planning and response assessment (46).

## 7 Treatment adaptation and patient outcome

PET imaging increasingly supports adaptive treatment paradigms by enabling response-based modulation of radiotherapy and radioligand therapy. In radiotherapy, mid-treatment PET (e.g., ^18^F-FDG PET) can guide dose adaptation: reductions or increases in metabolic activity and volumetric parameters during therapy may justify shrinking boost volumes for responders or escalating dose in PET-hot, potentially radioresistant sub-volumes. Evidence for this approach comes from non-small-cell lung cancer (NSCLC), where personalized treatment planning incorporating baseline and mid-treatment PET data reduced the risk of radiation pneumonitis while enabling tailored tumor dose escalation or normal tissue sparing (47).

In head-and-neck squamous cell carcinoma (HNSCC), early changes in tumor heterogeneity on mid-treatment FDG-PET, independent of HPV/p16 status, were associated with locoregional control: patients whose metabolic heterogeneity decreased showed markedly better 2-year locoregional recurrence-free survival compared with those without such decrease (48). Similarly, in esophageal cancer, relative changes in metabolic tumor volume (MTV) during chemoradiotherapy correlated significantly with locoregional recurrence and distant metastasis risk (49). These data suggest that mid-treatment PET metrics—beyond conventional SUV changes—may stratify patients by risk and allow truly adaptive radiotherapy, potentially improving efficacy while limiting toxicity.

In the context of radiopharmaceutical therapy (RPT), and especially in prostate cancer, PET-guided approaches are influencing outcomes. For instance, in recurrent prostate cancer after radical prostatectomy, salvage radiotherapy guided by PSMA PET/CT led to significant rates of biochemical recurrence-free survival (bRFS), effectively allowing many patients to defer long-term androgen deprivation therapy or systemic treatment (50).

More recently, in patients undergoing PSMA-targeted RPT, baseline PET parameters—particularly whole-body tumor SUV_mean—emerged as strong predictors of radiographic progression-free survival (PFS) and overall survival (OS), reflecting that quantification of tumor burden and heterogeneity via PET adds prognostic insight beyond PSA or conventional imaging alone (51).

Overall, these early clinical data demonstrate the feasibility and potential benefits of PET-driven adaptive strategies. Such approaches may enable enhanced locoregional control in external-beam radiotherapy, improved progression-free survival in RPT, reduced side effects via personalized dosimetry, and more efficient stratification of patients for combined or sequential therapies. Nevertheless, robust randomized trials remain scarce; most evidence derives from prospective cohorts or feasibility studies, and consistent standardization of PET parameters and adaptation criteria is still lacking (52).

## 8 Recommendations for clinical implementation

To effectively integrate PET into routine radiotherapy (RT) and radiopharmaceutical therapy (RPT) workflows, centers should begin by adopting standardized PET protocols that define key parameters such as time-post-injection, acquisition and reconstruction settings. Consistent imaging protocols are foundational, since quantitative PET metrics (e.g., SUV, metabolic tumor volume, and heterogeneity features) are highly sensitive to technical and biological variability; without standardization, inter- and intra-site differences may obscure true biological changes (53).

Where feasible, PET should be integrated early in therapy planning. A baseline or “simulation” PET (ideally acquired in radiotherapy treatment position) allows optimal target delineation and accurate co-registration with planning CT, forming a robust starting point for both EBRT and RPT (54). For centers aiming at advanced RT or adaptive strategies, PET-CT systems should be commissioned specifically for radiotherapy planning; this includes acceptance testing, ongoing quality assurance, and integration into the RT planning workflow—ideally within a broader QA framework overseen by medical physics experts (55).

Quantitative imaging further demands harmonized metrics. Harmonization programmes such as the accreditation under EANM Research Ltd. (EARL) provide validated methods to standardize SUVs, metabolic volumes, and other PET readouts across different PET/CT systems and centers (56), Use of EARL-compliant or otherwise harmonized reconstruction/analysis pipelines ensures that quantitative PET data remain comparable between patients, over time, and across studies or treatment cycles (57).

For RPT and dose-based planning, implementation of PET-based dosimetry frameworks is strongly recommended. Centers should adopt workflows that allow voxel-wise dosimetry, including multi-time-point imaging where needed, and document absorbed doses as recommended by consensus guidelines (58). Such dosimetry-based personalization may improve therapeutic index, reduce toxicity, and optimize combined EBRT/RPT strategies.

Successful deployment of these advanced imaging-based treatment paradigms requires a multidisciplinary approach. Collaboration between nuclear medicine specialists, radiation oncologists, medical physicists and dosimetry experts is essential for protocol development, image acquisition and reconstruction, image registration, contouring, dosimetry computations and treatment planning (55). Early PET-guided treatment planning is best implemented within the context of regular tumor boards or multidisciplinary treatment meetings where imaging, dosimetry and oncology decisions can be aligned.

Finally, centers are encouraged to participate in multicenter standardization and harmonization initiatives—for example through accreditation programs and data sharing frameworks—to facilitate pooling of data, cross-center comparability, and collective validation of PET-guided workflows (59).

## 9 Challenges, limitations and future directions

Despite the promise of PET-guided radiation therapy (RT) and radiopharmaceutical therapy (RPT), several substantial challenges impede widespread clinical adoption. A core issue lies in the heterogeneity of imaging protocols and quantification methods. PET images often suffer from limited spatial resolution, noise, and partial-volume effects, which impair accurate delineation of small lesions or micrometastases, complicate target-volume definition, and reduce quantitative accuracy for dosimetry calculations (60).

Segmentation of tumors and normal tissues on PET remains problematic: a variety of methods (manual contouring, thresholding, edge detection, gradient-based delineation, or more advanced algorithms) have been used, but no consensus exists on the optimal approach. These inconsistencies lead to large inter-observer and inter-center variability in target volumes, which undermines the reproducibility and reliability of PET-based planning (61). In lung cancer for instance, motion artifacts, respiratory movement, and mismatch between PET and CT during respiratory cycles may introduce registration errors and displace lesion boundaries by more than 10 mm—a significant challenge for radiotherapy planning (62).

Beyond technical and image-processing hurdles, implementation of personalized dosimetry in RPT faces practical and logistical bottlenecks. Accurate dosimetry requires high-quality quantitative imaging (PET or quantitative SPECT), multiple time-point imaging for pharmacokinetics, calibration of imaging systems, skilled medical physics input, and time-consuming segmentation and dose calculations. Many centers lack the infrastructure, personnel, training or financial reimbursement to carry out these tasks routinely (63). As a result, empiric fixed-activity regimens remain common—although they risk undertreating many patients whose individual biokinetics and tumor burden would permit more aggressive, yet safe, dosing (64).

The emerging field of PET-based radiomics and AI-driven image analysis brings further opportunities—but also new challenges. While radiomics promises to capture tumor heterogeneity and biological behavior beyond simple metrics such as SUV, current studies are often retrospective, single center, and lacking external validation. The reproducibility of radiomic features across different scanners, acquisition settings, segmentation and reconstruction pipelines remains poor (65). Without rigorous standardization and prospective multicentre validation, claims that particular radiomic signatures predict response, toxicity, or guide adaptation remain speculative (66).

Moreover, for newer theranostic approaches—including use of alpha-emitters, multi-target PET ligands, or combined modality treatments (e.g., RPT + EBRT, or PET-guided immunotherapy)—the current lack of robust radiobiological models is a major limitation. Even when dosimetry is performed, translating absorbed dose into biologically effective dose, predicting combined-modality effects, or anticipating normal tissue toxicity remains difficult. As noted in a recent conceptual study on “theranostic digital twins,” widespread implementation would require harmonized data collection, regulatory frameworks for data sharing, and clear ethical, legal and reimbursement paths (67).

Given these challenges, future progress will hinge on strong standardization efforts, multicenter cooperation, and technological innovation. Standardization of acquisition protocols, reconstruction parameters, segmentation, dosimetry workflows and post-processing is essential. Large-scale, prospective, multicentre trials should validate PET-based adaptive RT and RPT protocols, correlate PET-derived biomarkers with clinical outcomes, and define robust adaptation criteria. The development of advanced computational tools—including AI-based denoising, partial-volume correction, image reconstruction, radiomics feature extraction and even in-silico trials—may mitigate technical limitations and enable more precise, patient-specific planning (60).

In parallel, expansion of novel radiotracers and theranostic agents—including alpha-emitter therapies, multi-target PET ligands, and tracers for hypoxia, proliferation or immune microenvironment—may broaden the scope of PET-guided therapy. Exploiting high-sensitivity imaging modalities (such as total-body PET) to gather richer kinetic data may improve dosimetry accuracy and response modeling, especially for complex, individualized regimens combining EBRT, RPT, immunotherapy or other modalities. Recognizing these opportunities, the field must concurrently address regulatory, infrastructural, training and reimbursement barriers to ensure equitable and safe implementation.

## 10 Conclusion

PET imaging has fundamentally reshaped radiation oncology by providing biologically informed, quantitative, and personalized guidance for both external-beam radiotherapy (EBRT) and radiopharmaceutical therapy (RPT). Its applications span the entire treatment continuum, from patient selection and target delineation to dosimetry, therapy adaptation, and outcome prediction. By capturing tumor biology and heterogeneity, PET enables adaptive strategies that can modulate dose based on early response, optimize tumor coverage, and minimize normal tissue toxicity, thereby enhancing therapeutic efficacy and safety (47).

Recent technical advances, particularly in total-body PET, have dramatically increased sensitivity, enabling ultra-low-dose imaging, dynamic whole-body kinetics, and improved parametric quantification. These capabilities allow extended time-activity curve analysis for more accurate RPT dosimetry and support biologically adaptive radiotherapy decisions. (https://www.pmc.ncbi.nlm.nih.gov) In parallel, the development of novel PET biomarkers, such as fibroblast activation protein inhibitor (FAPI) tracers, and immune- or proliferation-targeted agents, provides additional avenues for precise tumor characterization, theranostic planning, and integration with EBRT or combined modality approaches (21).

The integration of PET-guided dosimetry frameworks, including voxel-wise RPT planning, together with standardized acquisition and analysis protocols, is central to translating biological information into clinically actionable treatment plans. Multidisciplinary collaboration between nuclear medicine, radiation oncology, and medical physics teams is essential to ensure accurate imaging, dose calculation, and adaptation strategies (53).

Conceptually and increasingly in early clinical experience, PET-guided radiation therapy represents a paradigm shift toward precision oncology, where therapy is driven by biology rather than solely by anatomy. Synergies between PET, EBRT, and RPT are increasingly recognized, offering the potential to refine patient selection, personalize treatment intensity, and improve clinical outcomes. To fully realize this promise, the field must continue to implement standardized imaging and dosimetry workflows, validate PET-based adaptive and combined modality approaches in prospective multicenter trials, and leverage emerging technologies such as total-body PET and novel tracers to optimize treatment personalization (53).

In summary, PET-guided therapy is transforming radiation oncology by providing a biologically informed, quantitatively robust, and patient-specific approach that integrates EBRT and RPT within a precision medicine framework, heralding a new era of biologically driven, outcome-oriented cancer care.
